# DIGITAL LITERACY, INTERGENERATIONAL RELATIONSHIPS, AND CARE PREPARATION AMONG CHINESE AGING ADULTS IN HONG KONG

**DOI:** 10.1093/geroni/igad104.1854

**Published:** 2023-12-21

**Authors:** Xue Bai, Wing-Lam Yu, Chang Liu

**Affiliations:** The Hong Kong Polytechnic University, Hong Kong, Hong Kong; The Hong Kong Polytechnic University, Hong Kong, Hong Kong; The Hong Kong Polytechnic University, Hong Kong, Hong Kong

## Abstract

The rapid ageing trend spotlights the need for more eldercare. Care preparation as a form of proactive coping can mitigate the negative effects of potential eldercare needs. Acquisition and preservation of resources are essential for effective proactive coping. The roles of digital literacy and intergenerational relationships as two significant resources in older people’s care preparation are still understudied. Integrating social convoy theory, intergenerational solidarity theory, and proactive coping theory, this study compared the levels of digital literacy, intergenerational relationships, and care preparation in son-dominant, daughter-dominant, and daughter & son-balance families; and examined the mediating effects of intergenerational relationships on the relationship between digital literacy and care preparation. Data from 3,626 participants with at least one adult child were drawn from the Panel Study of Active Ageing and Society, a biennial study conducted with a representative sample of people older than 50 years in Hong Kong. ANOVA was used to compare scores of key variables in three types of families. PROCESS was used to examine the mediation effects. Results showed that participants in a son-dominant family had the highest level of digital literacy; those in a daughter-dominant family reported the highest levels of intergenerational relationship quality and care preparation. Intergenerational relationship quality mediated the relationship between digital literacy and care preparation in three types of families while the effect sizes differed. These findings implicate the need to enhance older people’s digital literacy and foster their intergenerational relationships, thereby assisting them to be well-prepared for care needs in later life.

